# Evaluation of a Web-Based Food Record for Children Using Direct Unobtrusive Lunch Observations: A Validation Study

**DOI:** 10.2196/jmir.5031

**Published:** 2015-12-07

**Authors:** Anine Christine Medin, Helene Astrup, Britt Marlene Kåsin, Lene Frost Andersen

**Affiliations:** ^1^ Institute of Basic Medical Sciences Faculty of Medicine University of Oslo Oslo Norway; ^2^ Institute of Health and Society Faculty of Medicine University of Oslo Oslo Norway

**Keywords:** children, dietary records, Internet, observation, validity

## Abstract

**Background:**

High-quality, Web-based dietary assessment tools for children are needed to reduce cost and improve user-friendliness when studying children’s dietary practices.

**Objective:**

To evaluate the first Web-based dietary assessment tool for children in Norway, the Web-based Food Record (WebFR), by comparing children’s true school lunch intake with recordings in the WebFR, using direct unobtrusive observation as the reference method.

**Methods:**

A total of 117 children, 8-9 years, from Bærum, Norway, were recruited from September to December 2013. Children completed 4 days of recordings in the WebFR, with parental assistance, and were observed during school lunch in the same period by 3 observers. Interobserver reliability assessments were satisfactory. Match, omission, and intrusion rates were calculated to assess the quality of the recordings in the WebFR for different food categories, and for all foods combined. Logistic regression analyses were used to investigate whether body mass index (BMI), parental educational level, parental ethnicity or family structure were associated with having a “low match rate” (≤70%).

**Results:**

Bread and milk were recorded with less bias than spreads, fruits, and vegetables. Mean (SD) for match, omission, and intrusion rates for all foods combined were 73% (27%), 27% (27%), and 19% (26%), respectively. Match rates were statistically significantly associated with parental educational level (low education 52% [32%] versus high 77% [24%], *P*=.008) and parental ethnicity (non-Norwegian 57% [28%] versus others 75% [26%], *P*=.04). Only parental ethnicity remained statistically significant in the logistic regression model, showing an adjusted odds ratio of 6.9 and a 95% confidence interval between 1.3 and 36.4.

**Conclusions:**

Compared with other similar studies, our results indicate that the WebFR is in line with, or better than most of other similar tools, yet enhancements could further improve the WebFR.

## Introduction

High-quality dietary assessment tools are essential when studying children’s dietary practices. Traditional tools, such as food frequency questionnaires, 24-hour recalls, and food records, can be used to assess dietary intake [1-3]. In recent years, there has been a shift toward the use of Web-based dietary assessment tools among both adults and the younger age groups [4-6]; those aimed at children are mostly 24-hour recalls, or mixed methods combining food records and 24-hour recalls [7-12]. These new types of digital dietary assessment methods are highly needed [3].

In comparison with paper-based dietary assessment tools, Web-based tools facilitate data handling and improve user-friendliness; they reduce the burden for both the participant and researcher and can enhance motivation [3,6]. Therefore, we have recently adapted the Danish Web-based Dietary Assessment Software for Children (WebDASC) [7] to Norwegian conditions and food culture to develop the Web-based Food Record (WebFR) for children and adolescents.

It is well established that assessment of dietary intake is associated with errors [13]. Furthermore, assessing children’s intake is especially challenging due to their limited cognitive abilities [14], and because they often need assistance from a caretaker [13]. Consequently, validating dietary assessment tools that target children is important [1].

Direct observation is considered to be an appropriate high-quality method for validation studies of dietary assessment tools, because it provides direct unbiased information regarding what is eaten [15,16]. Hence, we aimed to evaluate the accuracy of children’s school lunch entries in the first Web-based dietary assessment tool for children in Norway, the WebFR, using direct unobtrusive observation as the reference method.

## Methods

### Participants

All the 4th graders (8-9 years old) from 4 elementary schools in Bærum, the fifth most populated municipality in Norway and a suburb of the capital city, were invited through the schools from September to December 2013. Convenience sampling was used; selected schools were in a short travel distance for the observers and had a highly cooperative school administration. Verbal and written information was given at plenary school meetings and in school classes to parents/guardians and children, respectively. To be included in the study, children needed an Internet access at their home, and their parents/guardians needed access to email. The final sample consisted of 117 of the 196 invited children (59.7%). The study was conducted in accordance with the Declaration of Helsinki. The Regional Ethical Committee in the South East of Norway found the study to fall outside their remit. Approval from the Norwegian Social Science Data Services was obtained, in addition to child assent and written parental consent from all participants.

### Design

The participants were instructed to record everything they ate and drank in the WebFR, for 4 consecutive days, including a weekend day. They were instructed to complete the recordings in the WebFR at home, with parental assistance, at the end of each recording day, after all meals were consumed. A practical demonstration was given at school in addition to written instructions on how to use the WebFR. During the days they recorded their diet, each child was observed once during school lunch. The children's weights and heights were also measured using standard procedures. After completing the study, the participants received a personal gift card with 2 cinema tickets.

### The Web-Based Food Record

**Figure 1:**
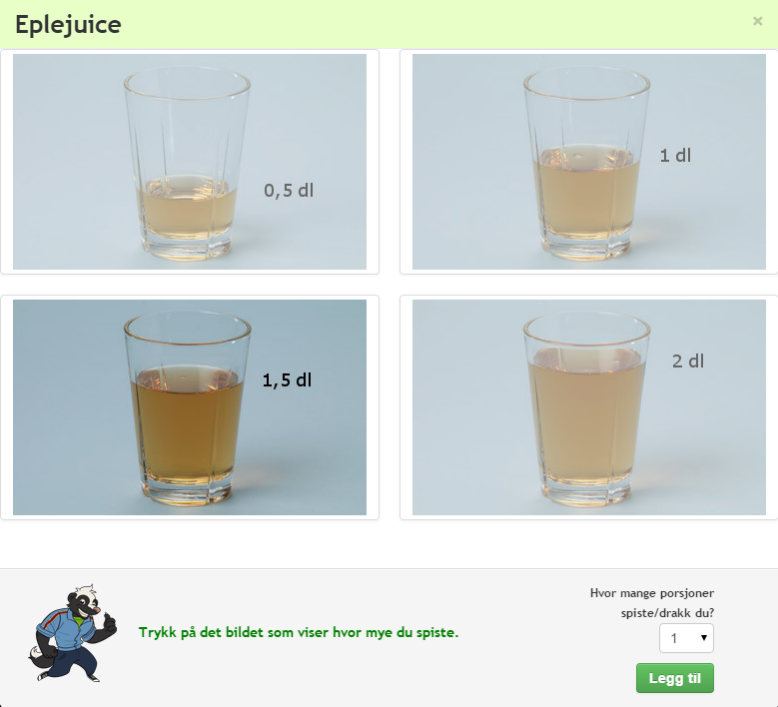
Screenshot from the Web-based Food Record (WebFR), showing an example of one of the photo series illustrating different portion sizes.

The WebFR is designed as a food record, yet including elements of a dietary recall, as recordings are completed by the end of each recording day. It is structured by meals with photos for portion-size assessments. It was adapted from the WebDASC by replacing its food lists with approximately 550 of the most commonly eaten foods and beverages in Norway, based on data from the latest Norwegian National Dietary Survey [17]. In addition, distinctive products designed for children (eg, children’s yoghurt) were also included based on Norwegian sales statistics. The photo series included both new photos taken specifically for the purpose of the development of the WebFR and selected photo series from the WebDASC, which were found appropriate to exemplify foods in the WebFR food list. Experienced dietitians considered the appropriateness of all the portion sizes included in the WebFR. All text and audio files were translated and slightly altered. The design of the interface was also changed to some extent; yet, the basic structure and functions of the WebDASC remained untouched. When recording, the participant selects the items consumed for each meal from drop down lists, or by using a free text search field. For each item, the participant chooses the most appropriate picture from a photo series with 2-4 photos illustrating different portion sizes, and then selects the number of portions eaten (Figure 1). Some of the photo series serve as proxies for other food items (eg, a glass of milk is illustrated by a glass of apple juice). When a food item is not found in the WebFR, it is possible to record it in an open field. A voice-assisted cartoon character guides the participant through the recordings. Prompts and pop-ups have been designed to ease recording in the WebFR and counter recall bias. Visuals of the WebFR are shown in Figure 1 and Multimedia Appendix 1.

### Observations

The observer team included 1 registered dietician and 2 master’s students in nutrition. The observations were performed in classrooms in which the children ate their home-packed lunches during regular school days. Each child was observed one time, during the same period as when they were instructed to record data in the WebFR. Each observer monitored a maximum of 3 children at the same time in an unobtrusive manner (ie, avoiding interaction with the participants and blinding the observations for participants). The children were already familiar with the presence of the observers prior to the observations, through instructional sessions.

The observers used a standardized form to take notes during their observations. To ensure complete recordings, observers were present in the classroom from before the children started eating to until they all had stopped eating. Immediately after each observation session, the observers categorized all observed food items into categories and portion sizes that corresponded to the information in the WebFR, with the aid of tablets containing the lists of categories, items, and all photos found in the WebFR. When the observed foods were not found in the WebFR, the observers described the food item in detail in text and chose the food category and portion size they considered most appropriate for the specific food item. After completion of the data collection, the observer team determined what constituted matches, omissions, and intrusions, using a strict definition; that is, a match was considered a match only when the child and observer clearly described the same item.

Observer training prior to data collection was conducted over a period of 3 weeks, based on the training protocol by Richter et al [18]. Interobserver reliability (IOR), which was expressed as the proportion of agreeing observations between observer pairs, was assessed during the training period and continuously during data collection. The overall agreement of observed food items was 92%, ranging from 88% to 96% between the 3 observer pairs. Lower agreement was found for observed portion sizes, with an overall agreement of 81% and a range from 77% to 88% between observer pairs.

### Variables

Variables for “matches,” “omissions,” and “intrusions” were created by comparing the observational data with the participants’ school lunch recordings in the WebFR. Matches are items that are both observed as eaten and recorded as eaten by the child; omissions are items that are observed as eaten but not recorded as eaten; and intrusions are items not observed as eaten, but recorded as eaten by the child.

Participants’ height and weight were measured according to standard procedures, without shoes and in light clothing, to the nearest millimeter and 0.1 kg, respectively, by trained personnel. A digital scale was used (TANITA TBF-300, Tanita Corporation, Tokyo, Japan), in the privacy of a separate room, for each participant. Age and sex-specific body mass index (ISO-BMI) cutoffs defining overweight and obesity among the study participants were applied [19].

Parents/guardians provided information in the written consent form regarding each participant’s sex and age, parental education level (low, intermediate, or high), parental ethnicity (at least one versus no parents/guardians of Norwegian origin), and family structure (mother and father of participant living in same household versus other).

### Statistical Analyses

MS Excel (version 2010, Microsoft, Redmond, WA, USA) was used to create all the variables. IBM SPSS (version 21.0, 2012, IBM Corp, New York, NY, USA) was used in all analyses, with the exception of the bias-reduced logistic regression analysis, for which the statistical package R (version 3.0.1, 2013, The R Foundation for Statistical Computing, Vienna, Austria) was used.

Descriptive statistics for the observed food items, recorded food items, matches, omissions, and intrusions were performed. The rates of matches, omissions, and intrusions were calculated for each participant both for all food items combined and at the food item category level (eg, “fruit, berries,” “bread products”). Definitions of these variables are in accordance with previous definitions developed by Baxter et al [20], but without using weighted values, ie, all food items were given equal statistical weight, and thus equal importance, in this study. “Coinciding omissions and intrusions” were also calculated, that is, cases in which a participant has an omission that corresponds to an intrusion within the same food category and within the same meal (eg, “apple” omitted and “pear” intruded during the same school lunch). The portion sizes of the omitted and intruded food items were counted using 4 different categories as follows: extra small (XS), small (S), medium (M), and large (L), based on the available photo series in the WebFR. Unclear observations of food items or portion sizes were excluded from analyses, such as the amount of water consumed from an opaque drinking bottle.

Univariate analyses were conducted to find possible differences in the match rates, omission rates, and intrusion rates as continuous variables, for all foods combined, with regard to the following variables: sex, BMI category, parental educational level, parental ethnicity, and family structure. Parametric tests were used when appropriate. Because the omission rate is the inverse of the match rate (match rate=100 - omission rate), testing for the match rate was therefore equivalent to testing for the omission rate.

A log transformation of the match rate variable was conducted; nevertheless, the assumptions for doing a multivariate linear regression were not present. Hence, match rates were further recoded to a dichotomous variable, which was defined as either a “low match rate” (≤70%) or “high match rate” (>70%). Logistic regression analyses were used to investigate the association between participant characteristics and the quality of the recordings in the WebFR (ie, low versus high match rate). Because of low cell counts, Logistf (bias-reduced logistic regression, Firth correction) [21] was also conducted in the statistical package R to compare the results with those from the logistic regression. The reporting in this study is in line with the STROBE guidelines [22].

## Results

The characteristics of the study sample are shown in Table 1. A total of 15 of 117 participants (12.8%) were overweight or obese. Furthermore, the parental education level was high among 77 of 111 children (69.4%), and a great majority had at least one parent/guardian of Norwegian origin.

**Table 1:** Characteristics of participants (N=117) in a validation study of a Web-based Food Record in Norway.

Characteristics		n	%
**Age, years**			
	8	13	11.1
	9	104	88.9
**Sex**			
	Girls	64	54.7
	Boys	53	45.3
**ISO-BMI cutoff categories**			
	Normal weight	102	87.2
	Overweight or obese	15	12.8
**Parental education level** ^a^			
	Low^b^	12	10.8
	Intermediate^c^	22	19.8
	High^d^	77	69.4
**Parental Ethnicity** ^e^			
	At least one parent/guardian of Norwegian origin	105	91.3
	Both parents/guardians of ethnic origin other than Norwegian	10	8.7
**Family Structure** ^f^			
	Mother and father of participant living in same household	87	78.4
	Other	24	21.6

^a^Information from 111 participants was available for “parental education level.” Complete information on both parents/guardians was available from 108 participants; the 3 cases with missing information from 1 parent/guardian were included in the table based on the 1 available parent/guardian's educational level.

^b^Both parents/guardians' education was maximum high-school level.

^c^One parent/guardian's education was maximum high-school level, and the second parent/guardian's education was at university-college or university level.

^d^Both parents/guardians' education was at the university college or university level.

^e^Information from 115 participants was available for “parental ethnicity.”

^f^Information from 111 participants was available for “family structure.”

Table 2 shows omission rates and intrusion rates for all food items combined, and for categories of food items, listed in descending order from the most to the least frequently observed. The overall averages for the omission and intrusion rates were 27% and 19%, respectively. At the food category level, the average rates varied widely between categories; “bread products” and “milk” were both frequently eaten, and had the lowest omission rates at 5% and 6%, respectively. “Spreads” were eaten most frequently, but the degree of omissions was higher. Food items in the categories “fruit, berries” and “vegetables, salads” were the third and fourth most frequently eaten, also with a high degree of omissions. By contrast, “biscuits, buns, waffles, cakes, and candy” were eaten infrequently, but had the highest proportion of omissions (85%). For intrusions, “bread products” and “dinner leftovers” had the lowest rates, and “beverages, other” and “yogurt” the highest.

**Table 2:** Omission rate^a^ and intrusion rate^b^ within different food categories, listed in descending order from the most to the least frequently observed, for all 8- and 9-year old children (N=117) in a validation study of a Web-based Food Record in Norway.

	Omission rate %		Intrusion rate %		Coinciding omissions and intrusions^c^	
	N^d^	Mean (SD)	N^d^	Mean (SD)	N^e^	n (%)
All food items	117	27 (27)	117	19 (26)	136	18 (13.2)
Spreads	93	29 (43)	79	17 (33)	41	7 (17.1)
Bread products	95	5 (22)	97	7 (26)	5	3 (60.0)
Fruit, berries	42	39 (48)	36	25 (44)	22	1 (4.5)
Vegetables, salads	33	45 (49)	23	21 (39)	23	0 (0.0)
Milk	49	6 (24)	52	12 (32)	3	1 (33.3)
Beverages, other^f^	44	18 (39)	62	42 (50)	8	2 (25.0)
Dinner leftovers	17	33 (43)	14	7 (27)	7	0 (0.0)
Miscellaneous	17	44 (50)	12	21 (40)	8	1 (12.5)
Biscuits, buns, waffles, cakes, and candy	12	85 (31)	4	38 (48)	12	1 (8.3)
Yogurt	11	64 (50)	9	56 (53)	7	2 (28.6)

^a^Omission rate = omissions/observed eaten food items × 100 = omissions/(omissions + matches) × 100. Omission rates were calculated for each participant within the different food categories. Participants who were not observed eating foods within a certain category (eg, “fruit, berries”) were excluded from the analyses for this category, regardless of what was recorded eaten.

^b^Intrusion rate = intrusions/recorded eaten food items × 100 = intrusions/(intrusions + matches) × 100. Intrusion rates were calculated for each participant within the different food categories. Participants who did not record eating foods within a certain category (eg, “fruit, berries”) were excluded from the analyses for this category, regardless of what was observed eaten.

^c^Cases where a participant had an omission that corresponds to an intrusion, within the same food category and within the same meal. For example, “apple” omitted and “pear” intruded during the same school lunch. Formula used: coinciding omissions and intrusions/omissions × 100.

^d^Number of participants included in analyses.

^e^Number of food items included in analyses.

^f^Of all intruded “beverages, other” 96% are drinking water.

In addition, Table 2 shows that 18 of all 136 omissions (13.2%) were “coinciding omissions and intrusions”; this proportion varied greatly within the different food item categories. Out of the small number of omissions in the “bread products” category, 3 out of 5 (60%) were coinciding omissions and intrusions, thus most of the omissions in this category were minor errors (eg, white bread replaced by whole grain bread). By contrast, the categories “spreads,” “fruit, berries,” and “vegetables, salads” had low proportions of coinciding omissions and intrusions, and thus most of the omissions in these categories were food items that the participants simply did not record.

Omissions and intrusions of large portion sizes are considered to be more severe than the omission or intrusion of small portion sizes. In Table 3, the proportion of different portion sizes for omissions and intrusions is presented for all food items combined, and for each food category. Although all types of portion sizes were omitted overall, there was a trend toward omitting smaller portions. This was not the case for overall intrusions, in which the medium- and large-portion sizes intruded more often than the smaller ones.

**Table 3:** Proportion of different sizes of omitted^a^ and intruded^b^ food items during school lunch for all 8- and 9-year-old participants (N=117) in a validation study of a Web-based Food Record in Norway.

Items	N^d^	Proportion of different sizes^c^ of omitted food items, n (%)					N^f^	Proportion of different sizes^c^ of intruded food items, n (%)				
		XS	S	M	L	Missing^e^		XS	S	M	L	Missing^e^
All food items	136	28 (20.6)	29 (21.3)	21 (15.4)	22 (16.2)	36 (26.5)	91	9 (9.9)	24 (26.4)	30 (33.3)	28 (30.8)	—
Spreads	41	7 (17.1)	7 (17.1)	12 (29.3)	3 (7.3)	12 (29.3)	22	2 (9.1)	10 (45.5)	6 (27.3)	4 (18.2)	—
Bread products	5	—	1 (20.0)	1 (20.0)	3 (60.0)	0 (0.0)	7	—	0 (0.0)	5 (71.4)	2 (28.6)	—
Fruit, berries	22	10 (45.5)	5 (22.7)	0 (0.0)	3 (13.6)	4 (18.2)	12	2 (16.7)	2 (16.7)	3 (25.0)	5 (41.7)	—
Vegetables, salads	23	5 (21.7)	9 (39.1)	5 (21.7)	1 (4.3)	3 (13.0)	7	1 (14.3)	4 (57.1)	1 (14.3)	1 (14.3)	—
Milk	3	0 (0.0)	0 (0.0)	0 (0.0)	2 (66.7)	1 (33.3)	6	0 (0.0)	0 (0.0)	2 (33.3)	4 (66.7)	—
Beverages, other	8	0 (0.0)	0 (0.0)	0 (0.0)	0 (0.0)	8 (100.0)	26	2 (7.7)	8 (30.8)	9 (34.6)	7 (26.9)	—
Dinner leftovers	7	1 (14.3)	2 (28.6)	2 (28.6)	1 (14. 3)	1 (14.3)	1	1 (100)	0 (0.0)	0 (0.0)	0 (0.0)	—
Miscellaneous	8	1 (12.5)	0 (0.0)	1 (12.5)	2 (25.0)	4 (50.0)	3	0 (0.0)	0 (0.0)	3 (100.0)	0 (0.0)	—
Biscuits, buns, waffles, cakes, and candy	12	4 (33.3)	5 (41.7)	0 (0.0)	2 (16.7)	1 (8.3)	2	1 (50.0)	0 (0.0)	1 (50.0)	0 (0.0)	—
Yogurt	7	—	0 (0.0)	0 (0.0)	5 (71.4)	2 (28.6)	5	—	0 (0.0)	0 (0.0)	5 (100.0)	—

^a^Items observed eaten, but not recorded.

^b^Items recorded, but not observed eaten.

^c^Portion sizes were divided into the following categories: XS=extra small, S=small, M=medium, L=large, based on the photo series available for each food item.

^d^Number of omitted food items included in analyses.

^e^Portion size not possible to observe with certainty, that is, when participants drank from dark-colored drinking bottles or milk cartons, or when participants ate a sandwich where spreads were partially hidden because it was placed in between 2 slices of bread.

^f^Number of intruded food items included in analyses.

The very few omissions in the “bread products” and “milk” categories were mostly of large portion sizes, whereas the omitted portion sizes from “spreads” were mostly of medium sizes. By contrast, the majority of omitted items in the categories “fruit, berries” and “vegetables, salads” were of small portion sizes.

Along the same lines as the omissions, the few intrusions in the categories “bread products” and “milk” were all of medium or large sizes. In the categories “fruit, berries,” “vegetables, salads,” and “spreads,” intrusions occurred for all portion sizes.

Mean rates within subgroups are presented in Table 4. Children with normal weight tended to have lower omission rates, than their overweight or obese peers. The omission rates differed in a statistically significant fashion between the parental education levels; higher omission rates were associated with lower parental educational levels (*P*=.008). Furthermore, we found a statistically significant lower omission rate among children with at least one parent/guardian of Norwegian origin in comparison with children having both parents/guardians of ethnic origins other than Norwegian (*P*=.04). A lower omission rate was also observed among participants from homes in which the mother and father lived together, compared with children from homes with a different family structure.

**Table 4:** Match rate,^a^ omission rate,^b^ and intrusion rate^c^ within different subgroups among the 8- and 9-year-old participants (N=117) observed during school lunch in a validation study of a Web-based Food Record in Norway.

Variables		Total (N)	Match rate %		Omission rate %		Intrusion rate %	
			Mean (SD)	*P* ^d^	Mean (SD)	*P* ^d^	Mean (SD)	*P* ^d^
Total participants (N)		117	73 (27)		27 (27)		19 (26)	
**Sex**				.59		.59		.28
	Girls	64	71 (30)		29 (30)		22 (29)	
	Boys	53	76 (22)		24 (22)		16 (23)	
**ISO-BMI cutoff categories**				.44		.44		.80
	Normal weight	102	74 (27)		26 (27)		19 (26)	
	Overweight or obese	15	69 (27)		31 (27)		21 (28)	
**Parental education level** ^e^				.008		.008		.006
	Low^f^	12	52 (32)		48 (32)		40 (38)	
	Intermediate^g^	22	69 (31)		31 (31)		24 (32)	
	High^h^	77	77 (24)		23 (24)		15 (21)	
**Parental ethnicity** ^i^				.04		.04		.49
	At least one parent/guardian of Norwegian origin	105	75 (26)		25 (26)		19 (26)	
	Both parents/guardians of other ethnic origin than Norwegian	10	57 (28)		44 (28)		24 (27)	
**Family structure** ^j^				.08		.08		.86
	Mother and father of participant living in same household	87	75 (27)		25 (27)		20 (26)	
	Other	24	64 (29)		36 (29)		21 (31)	

^a^Match rate = matches/observed eaten food items × 100 = matches/(omissions + matches) × 100. Match rates were calculated for each participant, for all food items combined.

^b^Omission rate = omissions/observed eaten food items × 100 = omissions/(omissions+ matches) × 100. Omission rates were calculated for each participant, for all food items combined.

^c^Intrusion rate = intrusions/recorded eaten food items × 100 = intrusions/(intrusions+ matches) × 100. Intrusion rates were calculated for each participant, for all food items combined.

^d^ *P* value for comparison of groups. Analysis of variance and *t* test were used when applicable; if not, the nonparametric Mann-Whitney or Kruskal-Wallis test was used.

^e^Information from 111 participants was available for “parental education level.” Complete information on both parents/guardians was available from 108 participants; the 3 cases with missing information from 1 parent/guardian were included in the table based on the 1 available parent/guardian's educational level.

^f^Both parents/guardians' education was maximum high-school level.

^g^One parent/guardian's education was maximum high-school level, and the second parent/guardian's education was at the university college or university level.

^h^Both parents/guardians' education was at the university college or university level.

^i^Information from 115 participants was available for “parental ethnicity.”

^j^Information from 111 participants was available for “family structure.”

For intrusion rates, the differences between groups were not statistically significant, except for parental education wherein higher intrusion rates were associated with lower parental educational levels (*P*=.006).

The logistic regression model in Table 5 shows that parental ethnic background and parental education level were the most important variables associated with a “low match rate” (≤70%). Although the “low educational level” effect was reduced when adjusting for other variables, this variable was still borderline significant. Parental ethnicity was the single most important variable associated with a low match rate; if both parents/guardians had an ethnic background other than Norwegian, the odds for a “low match rate” (≤70%) were higher than if at least one parent/guardian was of Norwegian ethnicity. However, the confidence intervals were wide. The results from the Logistf (bias-reduced logistic regression, Firth correction), analyzed due to low cell counts, were consistent with the results from the logistic regression model. Thus, the standard logistic regression was kept as the final model.

**Table 5:** Variables associated with having a low match rate (≤70%) among 8- and 9-year-old children recording in a Web-based Food Record compared with unobtrusive school lunch observation in Norway.

Variables		n (%) of children		Odds ratio (95% CI)	
		Overall (N=111)	With low match rate (≤70%) (N=44)	Unadjusted (N=111)	Adjusted^a^ (N=111)
**BMI category** ^b^					
	Normal weight	96 (86.5)	36 (81.8)	1	1
	Overweight or obese	15 (13.5)	8 (18.2)	1.9 (0.6-5.7)	1.6 (0.4-5.4)
**Parental ethnicity** ^c^					
	Norwegian origin	101 (91.0)	36 (81.8)	1	1
	Non-Norwegians	10 (9.0)	8 (18.2)	7.2 (1.5-35.9)	6.9 (1.3-36.4)
**Parental education level**					
	High	77 (69.4)	25 (56.8)	1	1
	Intermediate	22 (19.8)	10 (22.7)	1.7 (0.7-4.6)	1.6 (0.6-4.5)
	Low	12 (10.8)	9 (20.5)	6.2 (1.6-25.1)	3.8 (0.9-17.2)
**Family structure** ^d^					
	Mother and father of participant living in same household	87 (78.4)	31 (70.5)	1	1
	Other	24 (21.6)	13 (29.5)	2.1 (0.9-5.3)	2.0 (0.7-5.3)

^a^Adjusted for all other variables in the model in a logistic regression analyses.

^b^ISO-BMI cutoffs applied.

^c^Both parents/guardians of ethnic origin other than Norwegian, compared with at least one parent/guardian of Norwegian origin (reference).

^d^Family structure defined as everything else but “mother and father of participant living in same household” (ie, other) compared with “mother and father of participant living in same household” (reference).

## Discussion

### Main Findings

We found that 8-9-year-old children on average had a match rate of 73%, an omission rate of 27%, and an intrusion rate of 19%, when comparing parental-assisted entries of school lunch data in a WebFR with unobtrusive observations. Mean omission and intrusion rates for different food categories varied greatly. Lower parental educational levels and a non-Norwegian background were associated with less accurate recordings, but this must be interpreted with caution because of the low numbers in these subgroups.

### Comparisons With Other Work

Only a few other validation studies of Web-based 24-hour recalls/records for children have used observation during school meals as a reference method. Among these studies are the one on the Automated Self-Administered 24-hour Recall-Kids-2012 (ASA24-Kids-2012) among 9-11-year olds by Diep et al [23] in the United States, the Food Intake and Physical Activity of School Children (CAAFE) study among 7-10-year olds by Davies et al [12] in Brazil, the Portuguese Self-Administered Computerised 24-h Dietary Recall (PAC24) study among 7-10-year olds by Carvalho et al [24] in Portugal, the WebDASC study among 8-11-year olds by Biltoft-Jensen et al [25] in Denmark, and the study of the modified Self-Administered Children and Infant Nutrition Assessment (SACINA) used among 6-8-year olds by Hunsberger et al [26] in Sweden.

Our results are not directly comparable with these validation studies, partly because the rates of matches, omissions, and intrusions were not calculated in the same way as they were in our study. Nonetheless, we assert that it is possible to interpret the direction of the findings; in the CAAFE and ASA24-Kids-2012 studies, lower agreement between the recordings in the Web-based assessment tool and observations of school lunch were reported than in our study. The CAAFE study had average rates of 44% matches, 30% omissions, and 26% intrusions [12], whereas the ASA24-Kids-2012 study had average rates of 37% matches, 35% omissions, and 27% intrusions [23]. Parental assistance during recordings was encouraged and accomplished for most participants in our study. This was not an option in the CAAFE and ASA24-Kids-2012 studies, although children could ask simple questions to the researchers during recording in these studies. The lack of parental/adult assistance may partly explain differences in the results between studies, and we argue that children at this age need help when recording, which is also proposed elsewhere [8,23,27-29]. Furthermore, a low parental educational level can somewhat explain the dissimilar results between studies; a low educational level was associated with poorer recordings in both the CAAFE study and our study, where 64% and 11% had a low parental educational level, respectively. In addition, in the CAAFE and ASA24-Kids-2012 studies, the children recorded entries after a 24-hour time lag, whereas in our study, the children were instructed to record their data within the same evening. This difference may explain why the match rate was higher in our study because it is demonstrated that omissions and intrusions in children’s dietary recalls increase as a function of time [30].

The PAC24 study shows results that are more in line with our results, despite the lack of parental/adult assistance during recording [24]. An explanation could be the broad definition of matches applied in the PAC24 study, in contrast to our study in which matches were defined in a stricter manner.

Because the WebFR is a Norwegian version of the Danish WebDASC, we expected the results to be consistent with the findings from the WebDASC validation study [25]. Surprisingly, Biltoft-Jensen et al [25] found 82% matches, 3% omissions, and 14% intrusions for total foods and beverages, which are remarkably better than in our study, and their rates of omissions were very low in comparison with our 27% omission rate. This large discrepancy cannot be explained by the fact that their calculations were performed slightly differently than those in our study. Age affects children’s dietary reports [13,14]. Thus, we argue that age may partly explain the differences between the studies because the children were on average a year older in the WebDASC study than those in this study. In addition, we suggest that reactivity may have been a larger problem in the Danish version than in ours because of their interactive observation style; children were instructed to unpack their packed lunches, separate items, open up sandwiches, and place them on a plate before a photograph was taken. In addition, questions were asked regarding food trading and earlier snacking from their packed lunch [25].

A very high reporting accuracy was reported in the small validation study (n=25) of SACINA by Hunsberger and co-workers; in their study, overall food matches ranged from 86% to 98% [26]. Although children in this study were only to recall 1 lunch meal eaten the previous day, assisted by a dietitian using the Web-based SACINA instrument providing photos and portion estimates, we believe this cannot explain why the accuracy was so contrastingly high compared to other studies.

Baxter et al [20,30-35] compared 9-year-old children’s dietary reports in the form of traditional recalls (not Web-based) with direct observations of school meals in several studies. In some of these studies, the rates of omissions and intrusions were calculated and presented in a way that is comparable with our study; the results demonstrate that the omission and intrusion rates varied widely, and that reporting accuracy was reduced when the time intervals between eating and reporting increased. Our results are consistent with or better than the majority of the studies by Baxter et al for “same day recalls.”

Only a few studies report the rates of omissions and intrusions for selected food subcategories that are comparable with our findings. Vegetables and sweets were reported as the most often omitted food items in the PAC24 study, whereas beverages were the most commonly intruded item [24]. This is in line with the high omission rates for “vegetables and salads” and “biscuits, buns, waffles, cakes, and candy,” and the high intrusion rate for “beverages, other” found in our study. Nevertheless, Biltoft-Jensen et al [25] reported remarkably lower omissions for fruits and vegetables than in our study. Once more, we argue that reactivity may have been a large problem in Biltoft-Jensen et al’s study, and may explain the discrepancy between the studies.

To our knowledge, we are the first to report on “coinciding omissions and intrusions,” and by doing so we add important knowledge as to whether the omissions and intrusions represent major errors, and not just slightly imprecise recordings. The food category “spreads” had a high omission rate, and most of the omissions were major errors, not “coinciding omissions and intrusions.” This discovery has already led us to improve the WebFR, by including tailor-made prompts for “spreads.”

Taking the portion sizes of the omitted and intruded food items into account is important because it provides a better understanding of whether these omissions and intrusions are of great concern or not. We observed high omission rates in the food categories “fruit, berries” and “vegetables, salads”; however, the portion sizes of these categories were mostly small in contrast to the portion sizes of omitted “spreads.” Thus, we argue that the omissions of spreads are more troublesome than the omissions of fruits and vegetables in our WebFR.

Lower parental education levels have been associated with a higher degree of misreporting among children in the form of underreporting, or both underreporting and overreporting [36-38]. As already described, both our findings and the results of the CAAFE study show that lower parental education was associated with more recording errors [12]. However, the picture is a bit unclear when searching the literature for associations between ethnic backgrounds and omissions or intrusions in dietary assessment tools for children. Baxter et al [30,35] reported that there were no statistically significant differences in rates with regard to ethnicity. Yet, Baranowski et al [39] reported that Hispanic children described more problems when using the Food Intake Recording Software System, an early version of the ASA24-Kids-2012, than other children. Our study is coherent with the latter; although we must emphasize that there were a limited number of participants who had both parents of non-Norwegian origin. A possible explanation for our findings may be that having an ethnicity different from the majority is a proxy for having a different food culture and perhaps literary difficulties, which may be a barrier, even though participants can enter any type of food into the WebFR using the open field option. Furthermore, although audio files were included to assist those with lower reading skills, the WebFR still requires knowledge of the Norwegian language, and thus errors during recording may occur more often among persons with language difficulties. Consequently, we suggest that children and/or parents or guardians with language difficulties should be identified and given extra instructions on how to use the WebFR in future studies. They may benefit from direct personal contact with someone from the research crew, to ensure that they understand what to do.

Studies indicate that underreporting among children increases as BMI increases [37,40,41]. Nevertheless, the odds for a “low match rate” (≤70%) were only slightly higher and not significantly different for overweight/obese than normal weight children in our study. We believe this nonsignificant result may be explained by lack of power.

### Strengths and Limitations

The use of direct unobtrusive observations is one of the strengths of this study, because these provide exact information about what is consumed, without affecting the recordings [42,43]. Furthermore, we have demonstrated that our observations of home-packed school lunches were satisfactory; the overall consistency between observers for food items was 92%, which is considered sufficient [15,16,44], and in line with other IOR assessments conducted during observations of home-packed lunches [18]. Lower agreement was seen for the observed portion sizes. The combination of thorough training sessions prior to the study start and IOR assessments before and continuously during the study was important, and it provides a premise for high internal validity.

The small number of individuals in some of the subgroups is a limitation of this study, as the preferable adjustment for cluster effects (school level) proved infeasible due to lack of established statistical methods. Hence, the point estimates in the logistic regression analysis should be interpreted with caution.

For practical reasons, observations were restricted to school lunches and to children in 4th grade (8-9 years). Thus, a limitation is that we do not know whether our findings can be extrapolated to other meals or age groups. In addition, our participants had more highly educated parents/guardians and were less overweight or obese, than the average Norwegian population in which 29% have higher education [45], and 16% of third graders are overweight or obese [46]. However, the proportion of participants with non-ethnic-Norwegian parents/guardians was 20% (see Table 4), which is higher than the 14% average in the general Norwegian population [47]. Despite these differences, this study was performed in a similar setting to the one intended for later use, that is, the next national dietary survey among children in Norway. This contributes to its external validity in a positive manner.

### Conclusion

We have demonstrated that 8-9-year-old children had a mean match rate of 73% when recording their food intake from school lunch, with parental assistance, in a WebFR. Some children had difficulties recording, but the mean results were better than what have been reported in most validation studies of other Web-based dietary assessment tools among children. The WebFR could be improved further by including additional prompts for high omission rate foods. We suggest that children and their parents/guardians with language difficulties should be given extra support and information about how to use the WebFR in future studies.

## Appendix

Multimedia Appendix 1 Selected screenshots from the Web-based Food Record (WebFR).

## Abbreviations

ASA24-Kids-2012: Automated Self-Administered 24-hour Recall-Kids-2012
BMI: body mass index
CAAFE: the Food Intake and Physical Activity of School Children
IOR: interobserver reliability
ISO-BMI: age and sex-specific body mass index
PAC24: the Portuguese Self-Administered Computerised 24-h Dietary Recall
SACINA: Self-Administered Children and Infant Nutrition Assessment
WebDASC: Web-based Dietary Assessment Software for Children
WebFR: Web-based Food Record

## References

[1] McPherson R, Hoelscher D, Alexander M, Scanlon K, Serdula M (2000). Dietary assessment methods among school-aged children: Validity and reliability. Prev Med.

[2] Thompson FE, Subar AF, Coulston AM, Boushey CJ, Ferruzzi MG (2008). Dietary assessment methodology. Nutrition in the Prevention and Treatment of Disease. 3rd edition.

[3] Thompson Frances E, Subar Amy F, Loria Catherine M, Reedy Jill L, Baranowski Tom (2010). Need for technological innovation in dietary assessment. J Am Diet Assoc.

[4] Forster H, Fallaize R, Gallagher C, O'Donovan CB, Woolhead C, Walsh MC, Macready AL, Lovegrove JA, Mathers JC, Gibney MJ, Brennan L, Gibney ER (2014). Online dietary intake estimation: The Food4Me food frequency questionnaire. J Med Internet Res.

[5] Christensen SE, Möller E, Bonn SE, Ploner A, Wright A, Sjölander A, Bälter O, Lissner L, Bälter K (2013). Two new meal- and web-based interactive food frequency questionnaires: Validation of energy and macronutrient intake. J Med Internet Res.

[6] Adamson A J, Baranowski T (2014). Developing technological solutions for dietary assessment in children and young people. J Hum Nutr Diet.

[7] Biltoft-Jensen A, Trolle E, Christensen T, Islam N, Andersen LF, Egenfeldt-Nielsen S, Tetens I (2014). WebDASC: A web-based dietary assessment software for 8-11-year-old Danish children. J Hum Nutr Diet.

[8] Baranowski T, Islam N, Douglass D, Dadabhoy H, Beltran A, Baranowski J, Thompson D, Cullen KW, Subar AF (2014). Food Intake Recording Software System, version 4 (FIRSSt4): A self-completed 24-h dietary recall for children. J Hum Nutr Diet.

[9] Vereecken C, Covents M, Maes L, Moyson T (2014). Formative evaluation of the dietary assessment component of Children's and Adolescents' Nutrition Assessment and Advice on the Web (CANAA-W). J Hum Nutr Diet.

[10] Foster E, Hawkins A, Delve J, Adamson AJ (2014). Reducing the cost of dietary assessment: Self-completed recall and analysis of nutrition for use with children (SCRAN24). J Hum Nutr Diet.

[11] Moore HJ, Hillier FC, Batterham AM, Ells LJ, Summerbell CD (2014). Technology-based dietary assessment: Development of the Synchronised Nutrition and Activity Program (SNAP). J Hum Nutr Diet.

[12] Davies VF, Kupek E, de Assis MA, Natal S, Di Pietro PF, Baranowski T (2015). Validation of a web-based questionnaire to assess the dietary intake of Brazilian children aged 7-10 years. J Hum Nutr Diet.

[13] Livingstone MB, Robson PJ, Wallace JM (2004). Issues in dietary intake assessment of children and adolescents. Br J Nutr.

[14] Livingstone MB, Robson PJ (2000). Measurement of dietary intake in children. Proc Nutr Soc.

[15] Simons-Morton BG, Baranowski T (1991). Observation in assessment of children's dietary practices. J Sch Health.

[16] Baglio ML, Baxter SD, Guinn CH, Thompson WO, Shaffer NM, Frye Francesca HA (2004). Assessment of interobserver reliability in nutrition studies that use direct observation of school meals. J Am Diet Assoc.

[17] The Norwegian Directorate of Health (2012). Norkost 3. A nationwide dietary survey among 18-70 year old men and women in Norway-11 [In Norwegian].

[18] Richter SL, Vandervet LM, Macaskill LA, Salvadori MI, Seabrook JA, Dworatzek PD (2012). Accuracy and reliability of direct observations of home-packed lunches in elementary schools by trained nutrition students. J Acad Nutr Diet.

[19] Cole TJ, Bellizzi MC, Flegal KM, Dietz WH (2000). Establishing a standard definition for child overweight and obesity worldwide: International survey. BMJ.

[20] Baxter Suzanne Domel, Thompson William O, Litaker Mark S, Guinn Caroline H, Frye Francesca H A (2002). Low accuracy and low consistency of fourth-graders' school breakfast and school lunch recalls. J Am Diet Assoc.

[21] Firth D (1993). Bias reduction of maximum likelihood estimates. Biometrika.

[22] von EE, Altman DG, Egger M, Pocock SJ, Gøtzsche PC, Vandenbroucke JP (2007). The Strengthening the Reporting of Observational Studies in Epidemiology (STROBE) statement: Guidelines for reporting observational studies. Lancet.

[23] Diep CS, Hingle M, Chen T, Dadabhoy HR, Beltran A, Baranowski J, Subar AF, Baranowski T (2015). The Automated Self-Administered 24-Hour Dietary Recall for Children, 2012 Version, for Youth Aged 9 to 11 Years: A validation study. J Acad Nutr Diet.

[24] Carvalho MA, Baranowski T, Foster E, Santos O, Cardoso B, Rito A, Pereira MJ (2015). Validation of the Portuguese self-administered computerised 24-hour dietary recall among second-, third- and fourth-grade children. J Hum Nutr Diet.

[25] Biltoft-Jensen A, Bysted A, Trolle E, Christensen T, Knuthsen P, Damsgaard CT, Andersen LF, Brockhoff P, Tetens I (2013). Evaluation of Web-based Dietary Assessment Software for Children: Comparing reported fruit, juice and vegetable intakes with plasma carotenoid concentration and school lunch observations. Br J Nutr.

[26] Hunsberger M, Pena P, Lissner L, Grafström L, Vanaelst B, Börnhorst C, Pala V, Eiben G (2013). Validity of self-reported lunch recalls in Swedish school children aged 6-8 years. Nutr J.

[27] Foster E, Hawkins A, Simpson E, Adamson AJ (2014). Developing an interactive portion size assessment system (IPSAS) for use with children. J Hum Nutr Diet.

[28] Baranowski T, Islam N, Baranowski J, Martin S, Beltran A, Dadabhoy H, Adame S, Watson KB, Thompson D, Cullen KW, Subar AF (2012). Comparison of a web-based versus traditional diet recall among children. J Acad Nutr Diet.

[29] Burrows TL, Martin RJ, Collins CE (2010). A systematic review of the validity of dietary assessment methods in children when compared with the method of doubly labeled water. J Am Diet Assoc.

[30] Baxter SD, Thompson WO, Davis HC, Johnson MH (1997). Impact of gender, ethnicity, meal component, and time interval between eating and reporting on accuracy of fourth-graders' self-reports of school lunch. J Am Diet Assoc.

[31] Baxter SD, Smith AF, Litaker MS, Guinn CH, Shaffer NM, Baglio ML, Frye Francesca HA (2004). Recency affects reporting accuracy of children's dietary recalls. Ann Epidemiol.

[32] Baxter SD, Thompson WO, Litaker MS, Guinn CH, Frye Francesca HA, Baglio ML, Shaffer NM (2003). Accuracy of fourth-graders' dietary recalls of school breakfast and school lunch validated with observations: In-person versus telephone interviews. J Nutr Educ Behav.

[33] Baxter SD, Guinn CH, Royer JA, Hardin JW, Mackelprang AJ, Smith AF (2009). Accuracy of children's school-breakfast reports and school-lunch reports (in 24-h dietary recalls) differs by retention interval. Eur J Clin Nutr.

[34] Baxter SD, Smith AF, Guinn CH, Thompson WO, Litaker MS, Baglio ML, Shaffer N, Frye F (2003). Interview format influences the accuracy of children's dietary recalls validated with observations. Nutr Res.

[35] Baxter SD, Thompson WO, Smith AF, Litaker MS, Yin Z, Frye Francesca HA, Guinn CH, Baglio ML, Shaffer NM (2003). Reverse versus forward order reporting and the accuracy of fourth-graders' recalls of school breakfast and school lunch. Prev Med.

[36] Lioret S, Touvier M, Balin M, Huybrechts I, Dubuisson C, Dufour A, Bertin M, Maire B, Lafay L (2011). Characteristics of energy under-reporting in children and adolescents. Br J Nutr.

[37] Rangan AM, Flood VM, Gill TP (2011). Misreporting of energy intake in the 2007 Australian Children's Survey: Identification, characteristics and impact of misreporters. Nutrients.

[38] Murakami K, Miyake Y, Sasaki S, Tanaka K, Arakawa M (2012). Characteristics of under- and over-reporters of energy intake among Japanese children and adolescents: The Ryukyus Child Health Study. Nutrition.

[39] Baranowski T, Islam N, Baranowski J, Cullen KW, Myres D, Marsh T, de MC (2002). The food intake recording software system is valid among fourth-grade children. J Am Diet Assoc.

[40] Baxter SD, Smith AF, Litaker MS, Guinn CH, Nichols MD, Miller PH, Kipp K (2006). Body mass index, sex, interview protocol, and children's accuracy for reporting kilocalories observed eaten at school meals. J Am Diet Assoc.

[41] Fisher J O, Johnson R K, Lindquist C, Birch L L, Goran M I (2000). Influence of body composition on the accuracy of reported energy intake in children. Obes Res.

[42] Baxter SD, Hardin JW, Smith AF, Royer JA, Guinn CH, Mackelprang AJ (2009). Twenty-four hour dietary recalls by fourth-grade children were not influenced by observations of school meals. J Clin Epidemiol.

[43] Smith AF, Baxter SD, Hardin JW, Guinn CH, Royer JA, Litaker MS (2007). Validation-study conclusions from dietary reports by fourth-grade children observed eating school meals are generalisable to dietary reports by comparable children not observed. Public Health Nutr.

[44] Simons-Morton BG, Forthofer R, Huang IW, Baranowski T, Reed DB, Fleishman R (1992). Reliability of direct observation of schoolchildren's consumption of bag lunches. J Am Diet Assoc.

[45] Statistics Norway (2013). Population's Level of Education.

[46] Norwegian Institute of Public Health. Division of Epidemiology (2014). Child Growth in Norway 2008-2010-2012. Height, Weight and Waist Circumference among 3rd Graders [In Norwegian].

[47] Statistics Norway (2014). Immigrants and Norwegian-Born to Immigrant Parents in 13 Municipalities [In Norwegian].

